# Supplementary material for: Efficient message passing for cascade size distributions
Source: Sci Rep. 2019 Apr 25;9:6561. doi: 10.1038/s41598-019-42873-9 (PMC6484029; doi:10.1038/s41598-019-42873-9)
Supplement: Supplementary file 1 — Supplementary material for [file 41598_2019_42873_MOESM1_ESM.pdf]

Supplementary material for 'Efficient message passing for cascade size distributions'

Rebekka Burkholz,[1,2*]

[1] Chair of System Design, ETH Zurich, Switzerland
[2] Department of Computer Science, ETH Zurich
[3] Harvard T.H. Chan School of Public Health, Biostatistics
Boston MA 02115 USA
*To whom correspondence should be addressed; E-mail: rburkholz@hsph.harvard.edu.

Theorem 1. *Let $G = (V, E)$ be a tree and R_i, R_i^c for $i \in V$ response functions defining a cascade model. The final cascade size distribution $p_\rho(t/N) = \mathbb{P}(T_r = t)$ is given by the result of a message passing algorithm ending in the root r, where at each node $n \in V$, the following computations are performed based on $p_{A_{c_i}}, p_{I_{c_i}}$ received from their children:*

Case $d_n = 1$ (leaves):

$$\begin{aligned}
&p_{A_n}(0,0) = \mathbb{P}(T_n = 0 \parallel s_p = 1) = \mathbb{P}(s_n = 0 \parallel s_p = 1) = 1 - R_n(1) \\
&p_{A_n}(1,0) = \mathbb{P}(T_n = 1 \parallel s_p = 1) = R_n(1) \\
&p_{A_n}(0,1) = \mathbb{P}(T_n = 0, s_n = 1 \parallel s_p = 0) = 0 \\
&p_{A_n}(1,1) = \mathbb{P}(T_n = 1, s_n = 1 \parallel s_p = 0) = R_n(0) \\
&p_{I_n}(1,0) = p_{I_n}(0,1) = 0 \\
&p_{I_n}(0,0) = \mathbb{P}(T_n = 0, s_n = 0 \parallel s_p = 0) = 1 - R(0) \\
&p_{I_n}(1,1) = \mathbb{P}(T_n = 1, s_n = 1 \parallel s_p = 0) = R(0).
\end{aligned} \tag{1}$$

A node with degree $d_n > 1$ receives as input the distributions $p_{A_{c_i}}$, $p_{I_{c_i}}$ corresponding to its

children. We define p_{A_n*} and p_{I_n*} as their 2-dimensional convolutions:

$$p_{A_n*}(t,f) := p_{A_{c_1}} **p_{A_{c_2}} ** \cdots **p_{A_{c_{d_n-1}}}[t,f]$$
$$p_{I_n*}(t,f) := p_{I_{c_1}} **p_{I_{c_2}} ** \cdots **p_{I_{c_{d_n-1}}}[t,f].$$

Note that we have $p_{A_n*}(t,a) = p_{I_n*}(t,a) = 0$ for $t < a$.

Case $d_n > 1$, $n \neq r$:

$$\begin{aligned}
&p_{A_n}(t,0) = \mathbb{P}\left(T_n = t, s_n = 0 \parallel s_p = 1\right) \\
&+ \mathbb{P}\left(T_n = t; s_n = 1; s_p = 1 \to s_n = 1 \parallel s_p = 1\right) \\
&= \sum_{a=0}^{d_n-1} p_{I_n*}(t,a)\left(1 - R_n^c\left(a+1\right)\right) + \sum_{a=0}^{d_n-1} p_{A_n*}(t-1,a) R_n\left(a+1\right) \\
&p_{A_n}(t,1) = \mathbb{P}\left(T_n = t, s_n = 1 \parallel s_p = 0\right) = \sum_{a=0}^{d_n-1} p_{A_n*}(t-1,a) R_n^c\left(a\right) \\
&p_{I_n}(t,0) = \mathbb{P}\left(T_n = t, s_n = 0 \parallel s_p = 0\right) = \sum_{a=0}^{d_n-1} p_{I_n*}(t,a)\left(1 - R_n^c\left(a\right)\right) \\
&p_{I_n}(t,1) = p_{A_n}(t,1),
\end{aligned} \tag{2}$$

At root r:

$$\begin{aligned}
\mathbb{P}\left(T_r = t\right) =& \mathbb{P}\left(T_r = t, s_r = 0\right) + \mathbb{P}\left(T_r = t, s_r = 1\right) \\
=& \sum_{a=0}^{d_r} p_{I_r*}(t,a)\left(1 - R_r^c(a)\right) + \sum_{a=0}^{d_r} p_{A_r*}(t-1,a) R_r^c(a)
\end{aligned} \tag{3}$$

Proof. In the first step, we show that the distributions p_{A_n} and p_{I_n} are calculated correctly. The formulas at the leaves follow immediately from the variable definitions. Let's focus on the case $d_n > 1$ further. The cascade size of a subtree is the sum of its childrens/ subtree cascade sizes and the state of the node n:

$$T_n = \sum_{i=1}^{d_i-1} T_{c_i} + s_n. \tag{4}$$

Yet, these random variables are not independent. Therefore, we cannot just take the convolution of their distributions. However, order-conditioning on the state s_n, the distributions of the

children subtrees become independent.

1) Let's first study the case $s_n = 0$, which is considered by the variables $I_{c_i} = (T_{c_i}, s_{c_i}) \parallel s_n = 0$. Their convolution $p_{I_n *}(t, a)$ corresponds to the probability that $\sum_{i=1}^{d_i-1} T_{c_i} = t$, while exactly a of the children are active assuming that $s_n = 0$. According to Equ. (4), $T_n = \sum_i T_{c_i} = t$ and we have to add the probabilities of all possible events $a = 0, \cdots, d-1$. Those have to be multiplied with the adequate response of n considering the state of s_p. For $s_p = 0$, the node n has to withstand a activation of neighbors and does so with probability $1 - R_r^c(a)$, while it withstands for given $s_p = 1$ the $a+1$ activations of neighbors with probability $1 - R_r^c(a+1)$.

2) The remaining case $s_n = 1$ is more complicated, as n can be involved in the activation of children. A_{c_i} has been defined so that r_{c_i} indicates whether c_i has been activated before n or afterwards. If c_i is active before n, we have $s_{c_1} = 1$ and only $T_{c_i} \parallel s_n = 0$ matters. Otherwise ($r_{c_i} = 0$), c_i is active or not after the activation of n, yet we have to regard $T_{c_i} \parallel s_n = 1$ in Equ. (4). However, this is not enough. We only count activation cases in which n really triggers the activation of c_i. The remaining ones are considered already by $r_{c_i} = 1$, where the activation would also happen without n. In conclusion, $p_{A_n *}(t, a)$ is the probability that $\sum_{i=1}^{d_i-1} T_{c_i} = t$, while exactly a of the children are active before the activation of n and can potentially trigger this activation of n. Therefore, the response of the node n is either given by $R_n(a+1)$ when the parent is supposed to cause the activation of n, as in the formula for $\mathbb{P}(T_n = t; s_n = 1; s_p = 1 \to s_n = 1 \parallel s_p = 1)$ (and $\sum_{i=1}^{d_i-1} T_{c_i} = t - 1$ because of $s_n = 1$, see Equ. (4)). Or the response is $R_n^c(a)$ when the parent is inactive ($s_p = 0$) as in $\mathbb{P}(T_n = t, s_n = 1 \parallel s_p = 0)$.

In the second step of the proof, we have to argue that the distribution of T_r is identical to the cascade size distribution. The main argument follows similar lines of reasoning as before. Only, the root has d_r instead of $d_n - 1$ children and has no parent state to consider. Two natural responses to activated children can be distinguished, $s_r = 0$ and $s_r = 1$. For $s_r = 0$, we have

$T_r \parallel s_r = 0 = \sum_{i=1}^{d_r} T_{c_i} + s_r = \sum_{i=1}^{d_r} (T_{c_i} \parallel s_r = 0)$ where the $(T_{c_i} \parallel s_r = 0)$ are independent and their distribution is given by $p_{I_n *}(t, a)$ with a of the children are active. The probability that the root does not become activated in this case is $1 - R_r^c(a)$.

For $s_r = 0$, again the children that are active before the activation or r have to be counted. These are a with probability $p_{A_n *}(t-1, a)$, while the root becomes active in response with probability $R_r^c(a)$ and adds one active node to the total number of active nodes $t = T_r = \sum_{i=1}^{d_r} T_{c_i} + s_n = t - 1 + 1$. $\square$

The final message passing algorithm (SDP) is just an application of Theorem 1. Convolutions are calculated with the help of 2-dimensional Fourier transformations $\mathcal{F}$ and their inverse $\mathcal{F}^{-1}$ using $p ** q = \mathcal{F}^{-1}(\mathcal{F}p\mathcal{F}q)$. In practice, these are approximated by 2-dimensional (inverse) Fast Fourier transformations (FFT) (performed on adequately zero padded distributions). The pseudocode is given by Algorithm 1.

Algorithmic complexity of SDP. Let's first focus on computations for one node n. $|T_n|$ denotes the number of nodes in the subtree rooted in n. The convolutions require in total $O(d_n(|T_n| + |T_n| \log(|T_n|)))$ computations: They consist of $O(d_n|T_n|)$ multiplications (in Fourier space),
$O(d_n|T_n| \log(|T_n|))$ for a 2-dimensional Fast Fourier Transformation (FFT) and two inverse FFTs (of p_{A_n} and p_{I_n}). Next, $O(d_n|T_n|)$ computations are needed to obtain the distributions p_{A_n} and p_{I_n} based on the convoluted children distributions by Eq. (2). In total, adding up all computation for N nodes, we thus have $O(\sum_{n=1}^{N} (d_n(|T_n| + |T_n| \log(|T_n|))))$. Yet, we have two options to reduce the run time: a) limit the accuracy of the cascade size distribution so that $|T_n|$ can be substituted by a constant C. For instance, p_ρ can be defined only on an equidistant grid of $[0, 1]$. In this case, we are left with $O(\sum_{n=1}^{N} d_n) = O(N)$ computations. b) We can parallelize the matrix times vector multiplications, the FFTs, and the computations for nodes, as

ALGORITHM 1: SDP

Input: Tree $G = (V, E)$, root r, $N = |V|$, maximal degree d_{max}, responses: $(\forall i \in V)$ R_i, R_i^c

Output: Final cascade size distribution p_{T_r}

Initialization:

$p_{A_n}, p_{I_n} \in [0,1]^{N+1} x\{0,1\}$, $p_{A_n*}, p_{I_n*} \in [0,1]^{N+1} x\{0,\cdots,d_{max}\}$

```
/* Convolutions of childrens' distributions:              */
```

Function $f(p_1, \cdots, p_d, d)$**:**

return $\mathcal{F}^{-1}\Pi_{i=1}^{d} p_i$

```
/* Message passing.                                       */
```

for ***leaves in parallel*** **do**

Compute p_{A_n}, p_{I_n} by Equ. (1);

Send $\mathcal{F}p_{A_n}, \mathcal{F}p_{I_n}$ to parent;

end

for ***nodes*** n***,*** $n \neq r$***, who have received messages by all children, in parallel*** **do**

$p_{A_n*} = f\left(\mathcal{F}p_{A_{c_i}}, d_n - 1\right)$; $p_{I_n*} = f\left(\mathcal{F}p_{I_{c_i}}, d_n - 1\right)$;

Compute p_{A_n}, p_{I_n} by Equ. (2);

Send $\mathcal{F}p_{A_n}, \mathcal{F}p_{I_n}$ to parent;

end

for ***root r*** **do**

$p_{A_r*} = f\left(\mathcal{F}p_{A_{c_i}}, d_r\right)$; $p_{I_r*} = f\left(\mathcal{F}p_{I_{c_i}}, d_r\right)$;

Compute p_{T_r} by Equ. (3);

end

return p_{T_r}

long as they are still in distinct subtrees. The matrix times vector multiplication parallelization leaves maximally $O(\log(|T_n|) + |T_n| \log(|T_n|))$ for each n left, while the parallelization of Fast Fourier transformations can further bring the computations in node n down to $O(d_n \log(|T_n|))$. In sequence, we have to compute only along paths from a leave to the root. The longest path is maximally $N/2$ so that we can end up with a worst case run time of $O(N \log(N))$. A combination of a) and b) usually leads to an algorithm with much smaller run time. usually $O(h)$, where h refers to the height of a tree. In the worst case (with a long path to the root), this can still require $O(N)$ computations.

Further numerical experiments

We look at further exemplary parameter choices to analyze the approximation quality of the proposed message passing algorithms. As a proof of concept, we first focus on the tree discussed in the main manuscript. Fig. 1 visualizes the average cascade size for the two studied cascade models (obtained by BP). BP as well as SDP are in principle exact in this setting. Despite rather broad and seemingly chaotic multi-modal shapes of the cascade size distribution, SDP matches simulation results perfectly. For orientation purposes, we also show phase diagrams for the average cascade size either computed by BP or LTA.

Yet, for the configuration model network and the corporate ownership network, both consisting of loops, we have to acknowledge the difficulty of approximations close to sudden regime shifts in the parameter space, i.e., when the average cascade size changes abruptly despite only small changes in the parameters. For the configuration model and TM, the parameters $(\mu, \sigma) = (0.18, 0.1)$ and $(\mu, \sigma) = (0.2, 0.1)$ are chosen exactly before and after a sudden regime shift and even the average cascade size is not well matched. The same applies to the corporate ownership network for $(\mu, \sigma) = (0.19, 0.1)$ and $(\mu, \sigma) = (0.22, 0.1)$. The main problem originates in the fact that the node failure probabilities in TDA are not approximated well by BP.

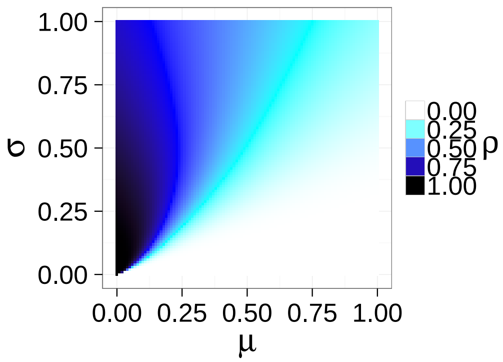

(a) Avg. ρ by BP for TM.

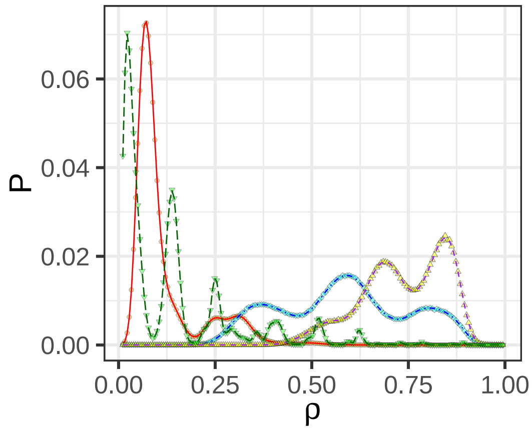

(b) SDP for TM.

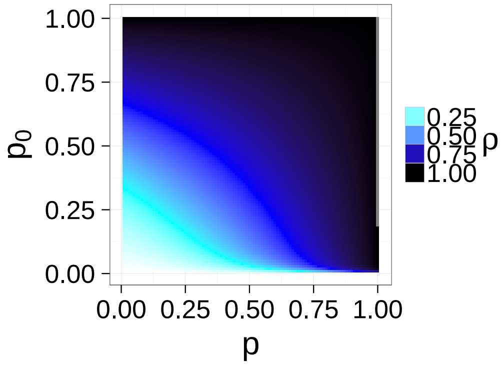

(c) Avg. ρ by BP for ICM.

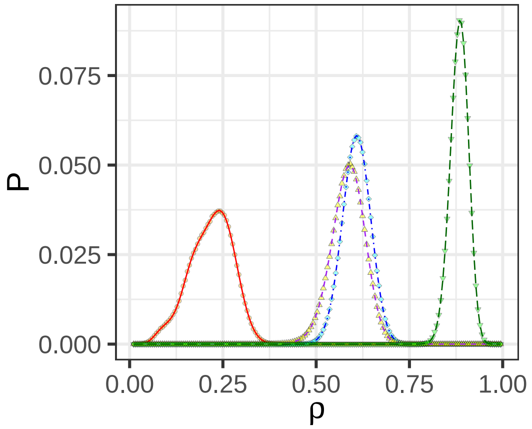

(d) SDP for ICM.

Figure 1: Tree. Left column: Average cascade size computed by Belief Propagation (BP) for the threshold model (TM) and independent cascade model (ICM). The thresholds are normally distributed by $F_i \sim \mathcal{N}(\mu, \sigma^2)$. For the ICM, nodes become active initially with probability p_0 and with probability p triggered by an active neighbor.
Right column: cascade size distribution for different parameters close and far away from the sudden regime shift. Symbols represent Monte Carlo simulations (with 10^6 realizations), lines result from SDP: orange circles/ red line ($(\mu, \sigma) = (0.75, 0.5)$), cyan squares/ blue line ($(\mu, \sigma) = (0.3, 0.5)$), yellow (upward pointing) triangles/ purple line ($(\mu, \sigma) = (0.1, 0.75)$), green (downward pointing) triangles / dark green line ($(\mu, \sigma) = (0.19, 0.1)$) and orange circles/ red line ($(p, p_0) = (0.2, 0.1)$), cyan squares/ blue line ($(p, p_0) = (0.2, 0.5)$), yellow (upward pointing) triangles/ purple line ($(p, p_0) = (0.5, 0.2)$), green (downward pointing) triangles / dark green line ($(p, p_0) = (0.5, 0.75)$).

Alternatives like a junction tree algorithm might provide better results, but is more computationally intensive and goes beyond this work. However, for all other parameters further away from a sudden regime shift, TDA approximates the cascade size distribution reliably.

Fig. 4 shows the final cascade size distribution for networks of increasing size. All have been generated by a configuration model with a degree sequence drawn from the same power law degree distribution $p(d) \sim d^{-2.5}$. We report results for the largest connected components with the number of nodes listed in the figure caption. The distributions that we receive take values in $\{0, 1/N, ..., 1\}$. To compare them on the same scale, we have downsampled the distributions to the support $\{0, 1/227, ..., 1\}$. First, we note that our algorithm scales well. For instance, we are able to compute the cascade size distribution for networks of $N \approx 30000$ nodes on a laptop (with 16 GB of RAM). Both, for the TM and the ICM, the mean squared error between the simulated empirical cascade size distribution and the TDA result decreases for increasing network size. Still, the convergence for the TM with $(\mu, \sigma) = (0.5, 0.5)$ is rather slow. Te probability at the peak is not matched perfectly, but the peak is correctly identified. The mean squared error for the largest network is $2.27 \cdot 10^{-9}$. The ICM leads to better results for the studied parameters. The mean squared error for the largest network is $3.05 \cdot 10^{-10}$.

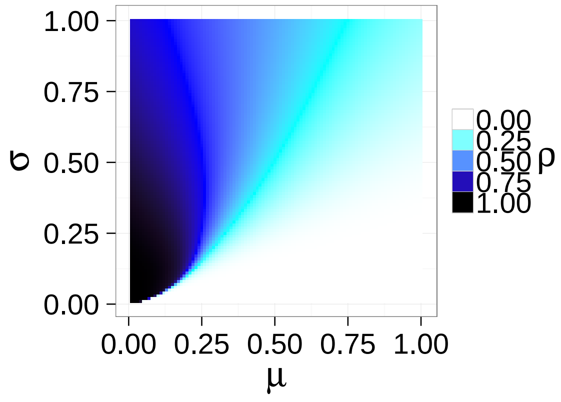

(a) Avg. ρ by BP for TM.

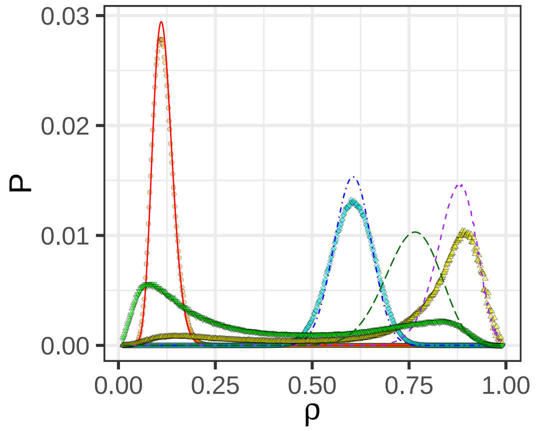

(b) TDA for TM.

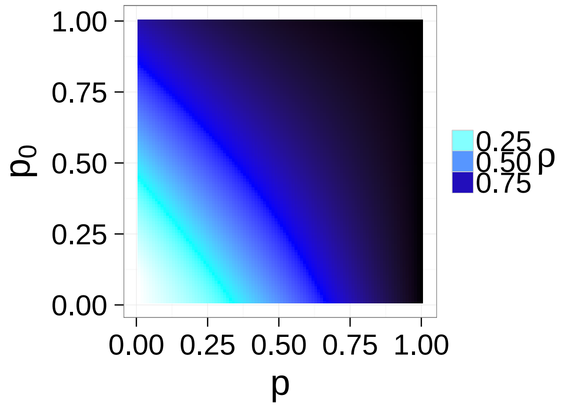

(c) Avg. ρ by BP for ICM.

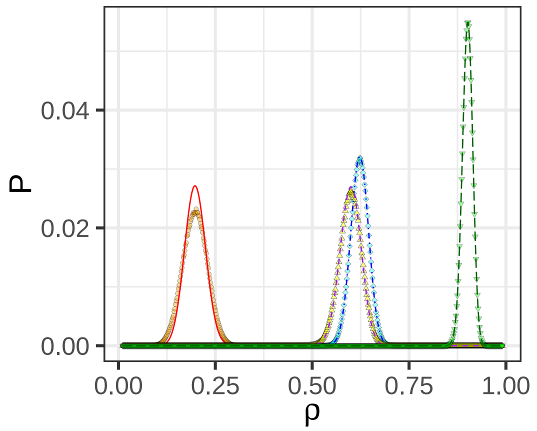

(d) TDA for ICM.

Figure 2: Configuration model network. Left column: Average cascade size computed by Belief Propagation (BP) for the threshold model (TM) and independent cascade model (ICM). The thresholds are normally distributed by $F_i \sim \mathcal{N}(\mu, \sigma^2)$. For the ICM, nodes become active initially with probability p_0 and with probability p triggered by an active neighbor.
Right column: cascade size distribution for different parameters close and far away from the sudden regime shift. Symbols represent Monte Carlo simulations (with 10^6 realizations), lines result from TDA: orange circles/ red line ($(\mu, \sigma) = (0.75, 0.5)$), cyan squares/ blue line ($(\mu, \sigma) = (0.3, 0.5)$), yellow (upward pointing) triangles/ purple line ($(\mu, \sigma) = (0.18, 0.1)$), green (downward pointing) triangles / dark green line ($(\mu, \sigma) = (0.2, 0.1)$) and orange circles/ red line ($(p, p_0) = (0.2, 0.1)$), cyan squares/ blue line ($(p, p_0) = (0.2, 0.5)$), yellow (upward pointing) triangles/ purple line ($(p, p_0) = (0.5, 0.2)$), green (downward pointing) triangles / dark green line ($(p, p_0) = (0.5, 0.75)$).

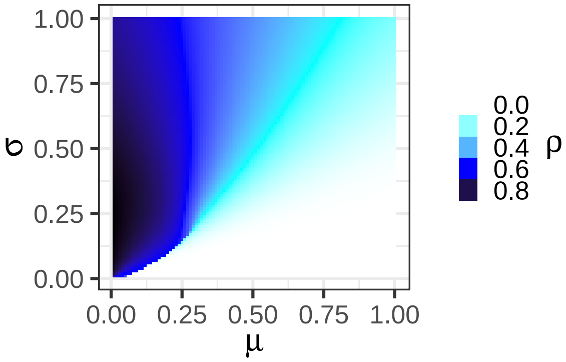

(a) Avg. ρ by LTA for TM.

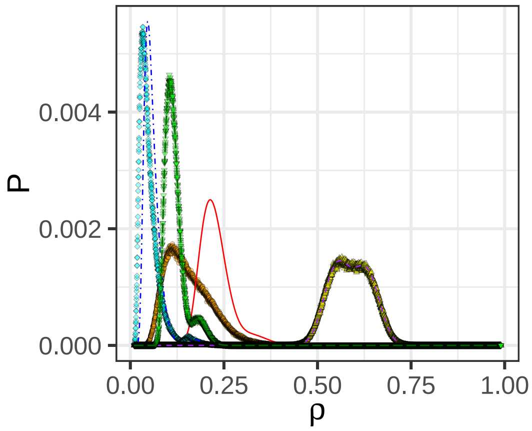

(b) TDA for TM.

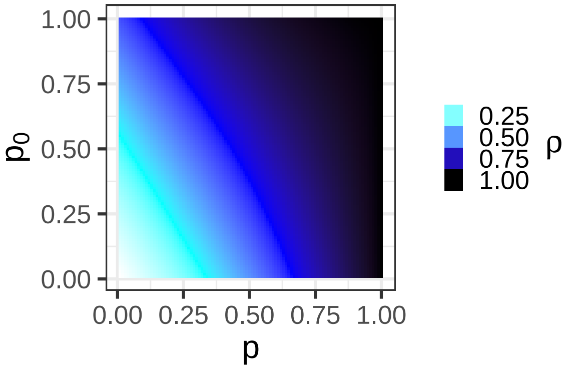

(c) Avg. ρ by LTA for ICM.

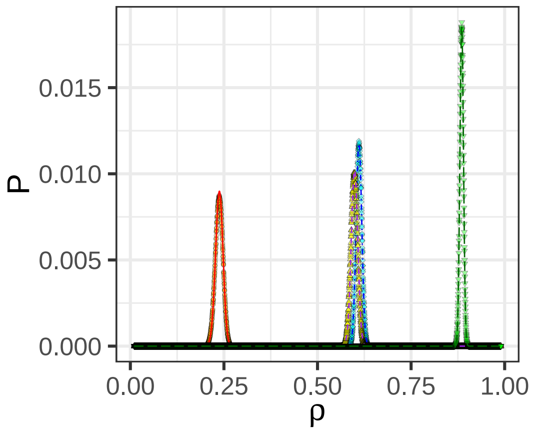

(d) TDA for ICM.

Figure 3: Corporate ownership network. Left column: Average cascade size computed by Local Tree Approximation (LTA) for the threshold model (TM) and independent cascade model (ICM). The thresholds are normally distributed by $F_i \sim \mathcal{N}(\mu, \sigma^2)$. For the ICM, nodes become active initially with probability p_0 and with probability p triggered by an active neighbor.
Right column: cascade size distribution for different parameters close and far away from the sudden regime shift. Symbols represent Monte Carlo simulations (with 10^6 realizations), lines result from TDA: orange circles/ red line ($(\mu, \sigma) = (0.19, 0.1)$), cyan squares/ blue line ($(\mu, \sigma) = (0.22, 0.1)$), yellow (upward pointing) triangles/ purple line ($(\mu, \sigma) = (0.3, 0.5)$), green (downward pointing) triangles / dark green line ($(\mu, \sigma) = (0.75, 0.5)$) and orange circles/ red line ($(p, p_0) = (0.2, 0.1)$), cyan squares/ blue line ($(p, p_0) = (0.2, 0.5)$), yellow (upward pointing) triangles/ purple line ($(p, p_0) = (0.5, 0.2)$), green (downward pointing) triangles / dark green line ($(p, p_0) = (0.5, 0.75)$).

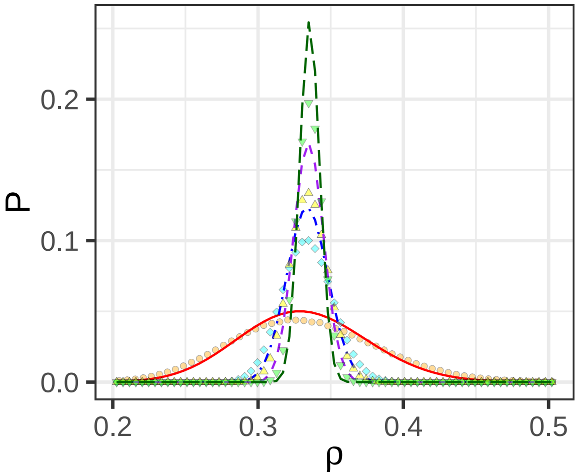

(a) TM $((\mu, \sigma) = (0.5, 0.5))$.

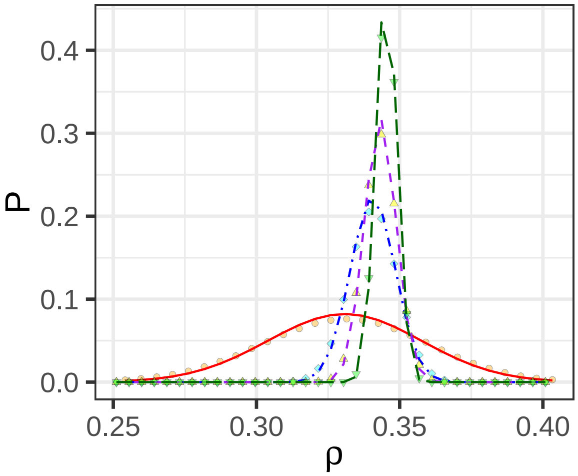

(b) ICM $(p, p_0) = (0.2, 0.2)$.

Figure 4: Configuration model networks with power law degree distribution $p(d) \sim d^{-2.5}$ of increasing number of nodes $N =$. Symbols represent Monte Carlo simulations (with 10^6 realizations), lines result from TDA: orange circles/ red line ($N = 543$), cyan squares/ blue line ($N = 5675$), yellow (upward pointing) triangles/ purple line ($N = 11557$), green (downward pointing) triangles / dark green line ($N = 29070$).
